# The Effect of Subcutaneous Epinephrine Dosage on Blood Loss in Surgical Incisions

**DOI:** 10.29252/wjps.9.3.309

**Published:** 2020-09

**Authors:** Seyed Esmail Hassanpour, Hatef Zirakzadeh, Yasaman Aghajani

**Affiliations:** 1 Department of Plastic and Reconstructive Surgery, Shahid Beheshti University of Medical Sciences, Tehran, Iran;; 2 Department of Anesthesiology; Khatam Al Anbia hospital, Tehran, Iran

**Keywords:** Epinephrine, Incision, Bleeding, Rat

## Abstract

**BACKGROUND:**

Epinephrine is commonly used in plastic and reconstructive surgeries to reduce the blood loss, and to achieveing the lowest and the most effective dosage of epinephrine can improve the results of the surgery.

**METHODS:**

Thirty two rats were divided in four groups. Local injection of epinephrine solution (3 mL) with concentrations of 1:200,000, 1:400,000 and 1:1,000,000 was undertaken in three different groups; and the fourth group was the control in which 3 mL of normal saline was administered 15 minutes before making the incision. The bleeding amount was compared in these groups.

**RESULTS:**

A significant difference in blood loss was noted between the control and other groups, but no significant difference was present between epinephrine concentrations of 1:200,000, 1:400,000 and 1:1,000,000.

**CONCLUSION:**

Local injection of epinephrine solution was shown to reduce bleeding from surgical incisions, but the difference between various pinephrine concentrations was not significant. The use of the lowest concentration (1:1,000,000) was suggested to decrease epinephrine side effects.

## INTRODUCTION

Surgical incision and skin damage is inevitable in surgeries, while bleeding due to these incisions happens. Bleeding is one of the most important complications in plastic surgery and injection of epinephrine has been widely used to decrease the amount of blood loss. Bleeding during surgery may cause complications, like hemoglobin decrease which may lead to blood transfusion.^1^^-^^3^ Bleeding has also adverse effects on wound healing process and aesthetic results and may cause cardiovascular and systemic side effects too. Wound bleeding can lead to more complications, especially in aesthetic and reconstructive surgeries, since these operations have longer surgical times and bleeding in surgical field may interfere with delicate surgery and anatomical plans on view.^4^^,^^5^

Some different methods have been used to decrease the bleeding amount during the operation such as controlled hypotensive state surgery and infiltration of vasoconstrictive drugs. All of these methods have their side effects and complications. Nowadays, vasoconstrictive drugs and especially subcutaneous epinephrine are commonly used in aesthetic and reconstructive surgeries to control bleeding.^4^^,^^6^ Epinephrine has been used in different doses for this purpose. It has local and systemic side effects and some of these side effects are severe cardiovascular complications, such as tachycardia, hypertension and even sometimes cardiac arrest and death. Epinephrine is used in concentrations from 1:100,000 to 1:1,000,000 and there is no consensus over standard dose of this vasoconstrictor for optimal results.^2^^,^^4^^,^^7^ Epinephrine side effects are dose dependent, and by decreasing epinephrine dosage, the side effects can decrease. In this study we assessed the effect of different doses of epinephrine on bleeding amount of surgical wounds in experimental rat model to suggest the optimal dosage of epinephrine to balance its effect on bleeding. 

## MATERIALS AND METHODS

**Fig. 1:**
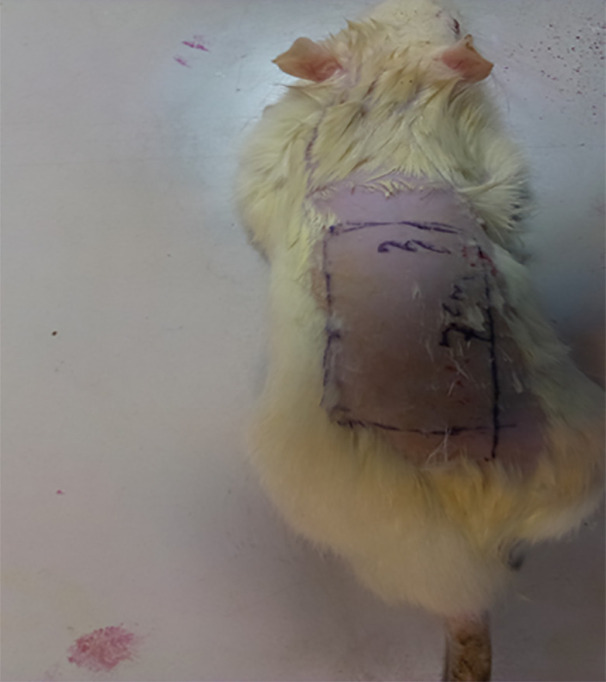
Designing the flap

This study is a randomized prospective study approach. Guidelines of the Declaration of Helsinki were followed in this research and the study was approved in the institution ethics committee. Thirty two healthy rats weighing 250 to 320 g (Mean: 300 g) were divided into four groups of 8 animals. Rats were anesthetized with 100 mg/kg of ketamine and 50 mg/kg of xylazine. The lumbar region of rats was shaved and a random pattern flap with 3×7 cm dimension was designed in this region (Figure 1). 

**Fig. 2:**
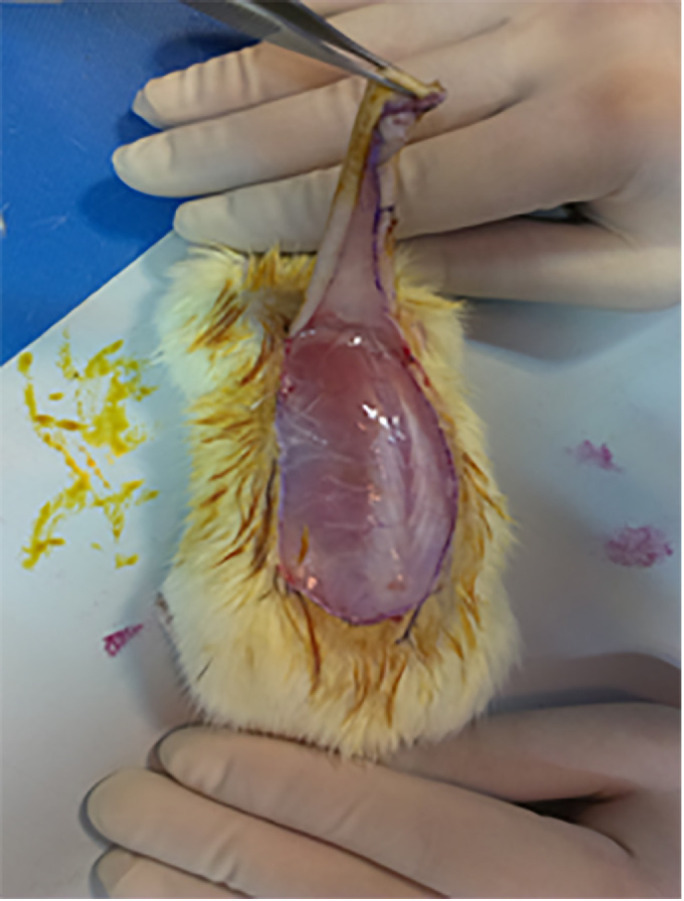
Elevating the flap

Three milliliters of epinephrine in normal saline with different concentrations of 1:200,000, 1:400,000 and 1:1,000,000 were injected subcutaneously under the designed flaps, before making the incision in three different groups of rats, while each group was consisted of eight rats. In the control group, only 3 mL of normal saline was used. Incisions were made 15 minutes after the injection and flaps were released (Figure 2). Bleeding was measured for 5 minutes by using 1×1 inch gauze. We weighed the gauze before making the incision and then weighed the gauze again after 5 minutes, when it was soaked with blood. We measured the weight of one milliliter of blood of rats and it was 0.95 g and we used this ratio to measure the blood loss. Analysis of data was done with SPSS software (version 16, Chicago, IL, USA). A p value less than 0.05 was considered statistically significant.

## RESULTS

The average amount of bleeding in 1:200,000, 1:400,000, 1:1,000,000 groups were 0.2525 mL, 0.2775 mL and 0.2638 mL, respectively and the difference between these groups was not significant (*p*>0.5). The average amount of bleeding in the control group was 0.5038 mL and had a significant difference with other groups (*p*<0.001). 

## DISCUSSION

This study illustrated that different concentration of epinephrine decreased the amount of bleeding from surgical incisions. Neither the lowest nor the highest epinephrine concentration could reduce bleeding amount from surgical field. All epinephrine concentrations resulted in same side effects.^8^^-^^10^ Different approaches have been tried to enhance vasoconstrictor effects, at the same time to reduce the dosage. Thus the optimum concentration of epinephrine for the prevention of bleeding has not been clearly resolute,^11^ we suggested to administer lower concentrations of epinephrine to decline the side effects, and perhaps 1:1,000,000 concentration has been the best dosage with the least complications. Epinephrine may also have an adverse effect on flap survival due to its vasoconstrictive effect and this will need further study.^11^

Another method to reduce epinephrine and local anesthetic concentration is to use them as combination this may also prolongs analgesia. To lessen the dosage of epinephrine, the lowest preferred dose should be administered to achieve vasoconstriction.^12^ Kim *et al.* suggested that epinephrine concentrations between 1:100,000 and 1:400,000 were similarly effective and provided better vasoconstriction when compared with more diluted solutions. If epinephrine is more diluted, its characteristics such as onset and time to peak serum concentrations may be influenced and also the duration of action is shortened.^13^

We have to consider that the optimum safe dose of local anesthetics is more dependent on the route, rate and the site of injection than the drug load.^13^ As mentioned before, site and sort of surgery affect optimum concentrations of epinephrine; while in dermatologic plastic surgeries, epinephrine in 1:50,000 concentration is used to reduce the amount of bleeding with satisfying results.^14^ The prevalence of cardiovascular toxic adverse effects has been observed to increase in a dose dependent manner.^15^^,^^16^ Many authors applied 1:200,000 concentration of epinephrine to control bleeding in aesthetics surgery, but we suggested administration of lower doses as described before.^6^ Yang and colleagues showed that before scalp incision in craniotomy, different concentrations of epinephrine have no significant effect on bleeding and this is compatible with our findings too.^6^

## CONCLUSION

We found that epinephrine can decrease bleeding from surgical incisions, but there was no different between 1:200,000, 1:400,000 and 1:1,000,000 concentrations. So we suggested choosing lower concentrations. 
